# A rapid review of periviable (22 + 0 to 23 + 6 weeks) counselling practices and the need for a trauma-informed care approach

**DOI:** 10.3389/fped.2025.1553040

**Published:** 2025-05-22

**Authors:** J. Peterson, C. Graham, E. D. Johnstone, A. Mahaveer, D. M. Smith

**Affiliations:** ^1^ Faculty of Biology, Medicine and Health Sciences, University of Manchester, Manchester, United Kingdom; ^2^ Neonatal Intensive Care Unit, St Mary’s Maternity Hospital, Manchester Foundation Trust, Manchester, United Kingdom; ^3^ Department of Paediatrics, Alder Hey Children's Hospital, Liverpool, United Kingdom; ^4^ Maternity Services, St Mary's Maternity Hospital, Manchester University NHS Foundation Trust, Manchester, United Kingdom

**Keywords:** neonates, extreme preterm, periviable, resuscitation, counselling, trauma and maternity care

## Abstract

Given the risks of mortality and morbidity for infants born in the periviable period, a decision is made between parents and professionals prior to the birth as to whether survival-focused or comfort care is most appropriate at delivery. Medical information should be shared with parents and parental perspectives and priorities in relation to this information should be explored and integrated into the decision-making process. Conducting these conversations is complex and nuanced. This rapid review conducted a systematic search of the available literature relating to the approaches to and content of the pre-birth periviable conversation and identified three core themes: Transparency, Collaboration and Empowerment. In brief, these themes demonstrate that the information provided to parents should consistently outline all available care options relevant to their baby, including compassionately delivered, but honest and descriptive accounts of emotive options, such as comfort care. Information should be individualised to the specific circumstances and risk factors of that individual family. Perinatal professionals should seek to incorporate discussion of topics key to the ‘good parent belief’ to empower parents within their role. Avoiding or omitting discussion of uncertainty and dismissal of hope within these conversations was associated with parental distrust and impaired communication. The themes identified within this rapid review align with the principles of trauma-informed care and provide a structure for further research and service development focused on improving the quality and experience of pre-birth periviable conversations for future parents.

**Systematic Review Registration**: https://www.crd.york.ac.uk/PROSPERO/view/CRD42022300099, PROSPERO identifier CRD4202230009.

## 1 Introduction

Neonatal medicine is a rapidly evolving field which has seen tremendous advances in care for infants born with a range of conditions over the last 60 years (1). Throughout that time optimising care of the preterm infant has been a priority and high yield area. As knowledge and technologies have progressed, the ‘periviable’ period (the point in a pregnancy where survival outside the womb is possible, even if improbable) has been continually adjusted (2) (3). Perinatal centres around the world are now able to provide care to infants from 22 weeks gestation with increasing chances of survival (4). Provision of survival-focused care to these infants requires a prolonged period of hospitalisation and all the inherent burdens that come with intensive care—for the infant, their parents, siblings and wider families. There are significant risks of death and disability for infants born in the periviable period, alongside lifelong physical health consequences and increased mental health impacts for families (5, 6). This is not to say that survival-focused care should not be provided to periviable infants. Rather - as is increasingly encouraged by professional frameworks—where possible, a holistic assessment of the individual infant should be made prior to delivery and a nuanced and honest conversation had between perinatal professionals and parents about whether survival-focused care or comfort care would be most appropriate (3). Conducting these conversations is complex and carries an emotional and moral burden for those involved (7). Professional frameworks, whilst encouraging shared decision-making approaches, may lack detail on how to navigate these conversations and meaningfully explore and facilitate incorporation of parental perspectives.

The periviable infant is now a topic of increasing research focus and funding. This has been seen through the expanding number of publications (Supplementary File S1) and incorporation of this topic within perinatal conferences (8–10). Our aim with this review is to succinctly outline the key findings from the currently available literature pertaining to information sharing practices between perinatal professionals and parents facing periviable birth and identify knowledge gaps for future research in this rapidly expanding field.

## 2 Methods

### 2.1 Why was a rapid review approach selected?

With the proliferation in academic and clinical interest in optimising care for periviable infants it is important that current research findings are distilled and summarised to enable practising clinicians to remain up to date with best practice management for the dilemmas encountered in managing periviable birth. Additionally, there is a need to provide systematic summaries to streamline identification of key knowledge gaps and provide areas of focus for future research. A rapid review approach was selected for this review to match the pace at which the area of periviable research is expanding, whilst retaining the rigor and transparency of a systematic review process, thus allowing perinatal professionals to have confidence in the review findings (11–13).

This review focuses on literature published from 2021 onwards. This date range was determined in several ways, through acknowledging significant changes in professional guidance, such as the updated framework from the British Association of Perinatal Medicine released in October 2019, an acknowledgement of the potential for COVID-specific publications in 2020 and, crucially, the notable expansion in publications related to periviable birth from 2021 onwards (Supplementary File S1). Given these factors and the need for this review to reflect up to date knowledge regarding information sharing practices with which to make recommendations, this review summarises literature published between 1st January 2021 to the date the search was conducted (29th July 2024).

The study protocol was developed in accordance with the Preferred Reporting Items for Systematic Reviews and Meta-Analyses (PRISMA) process (15). The study protocol was pre-registered with PROSPERO (CRD42022300099) (14).

### 2.2 Patient and public involvement

This rapid review has been conducted as part of a wider mixed methods study exploring information sharing practices between perinatal professionals and parents facing periviable birth [PeriviAble DeLiveries: ALIgning PArental aNd PhysiCian PrioritiEs (ALLIANCE)]. The Alliance study has received full approval and support from the Spoons Parental Advisory Group (PAG) who provide support to parents across the North West. When designing this study, members of our research team met with the PAG to gather their perspectives on the issue of periviable communication practices. This topic resonated with multiple parents who articulately outlined their own lived experiences citing numerous situations where they had been given either vague or directly conflicting information from perinatal professionals during their pre-delivery extremely preterm labours. Parents described how information was often either presented with a distressing inaccuracy and lack of empathy (“you're 22 weeks so there's nothing we can do”^1^), or, with such callously changing variability that parents lost confidence that the team were taking the life of their unborn child seriously. All parents in the PAG meeting agreed that the current level of variation in information provided to parents in labour is unacceptable and improving this should a priority issue for neonatal research agendas. This rapid review forms the first step in tackling this issue by reviewing the existing literature available to perinatal professionals and identifying key gaps and strategies to address these.

### 2.3 Ethical consideration

This study is a rapid review of previously published data and therefore, did not require formal ethics approval. All included studies had ethical approval in place and were conducted in accordance with the Declaration of Helsinki.

### 2.4 Search strategy

**Figure 1:**
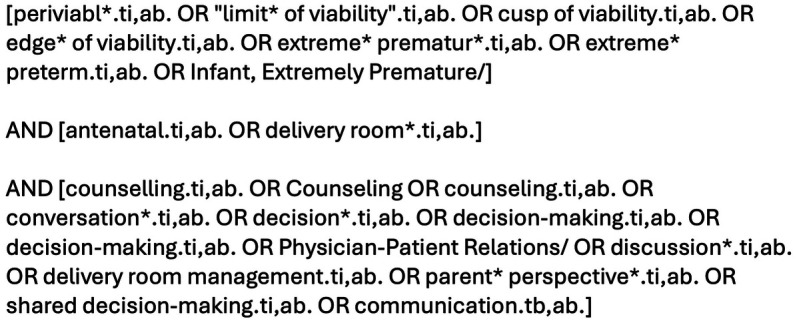
Search strategy.

**Figure 2:**
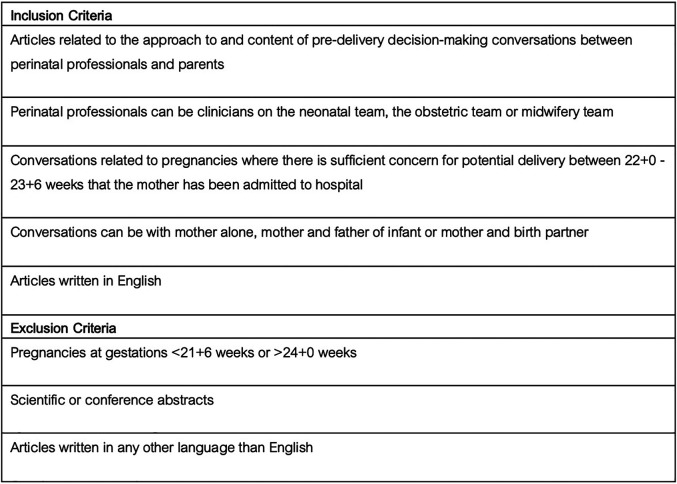
Inclusion/exclusion criteria.

The SPICE (Setting, Perspective, Intervention/phenomenon of Interest, Comparison, Evaluation) framework was used as a conceptualizing framework to formulate the review questions, keywords and search process (15, 16). The SPICE elements were outlined: Setting = Pregnancies at risk of delivery at periviable gestation (22 + 0 to 23 + 6 weeks); Perspective = Perinatal professionals involved in pre-delivery periviable delivery conversations with parents; Intervention (Phenomenon of interest) = The pre-delivery decision-making conversation; Comparison = No comparison; Evaluation = The approach to and the information topics included in pre-delivery decision-making conversations. Boolean operators were utilised to combine keywords and blocks. Additionally, the authors’ used the databases’ specific thesauri, truncation, and phrase searches to ensure the search was as comprehensive as possible. The search strategy was developed by JP and DMS with expert support from the hospital medical librarian. The search strategy (Figure 1) and inclusion/exclusion criteria (Figure 2) were agreed prior to conducting the search. The MEDLINE® (OVID) and Embase™ (OVID) databases were searched using the pre-agreed search strategy. Publications in English were eligible. Funding was not available for translation of articles published in other languages. The search was performed on 29th July 2024.

### 2.5 Study selection and critical appraisal

**Figure 3:**
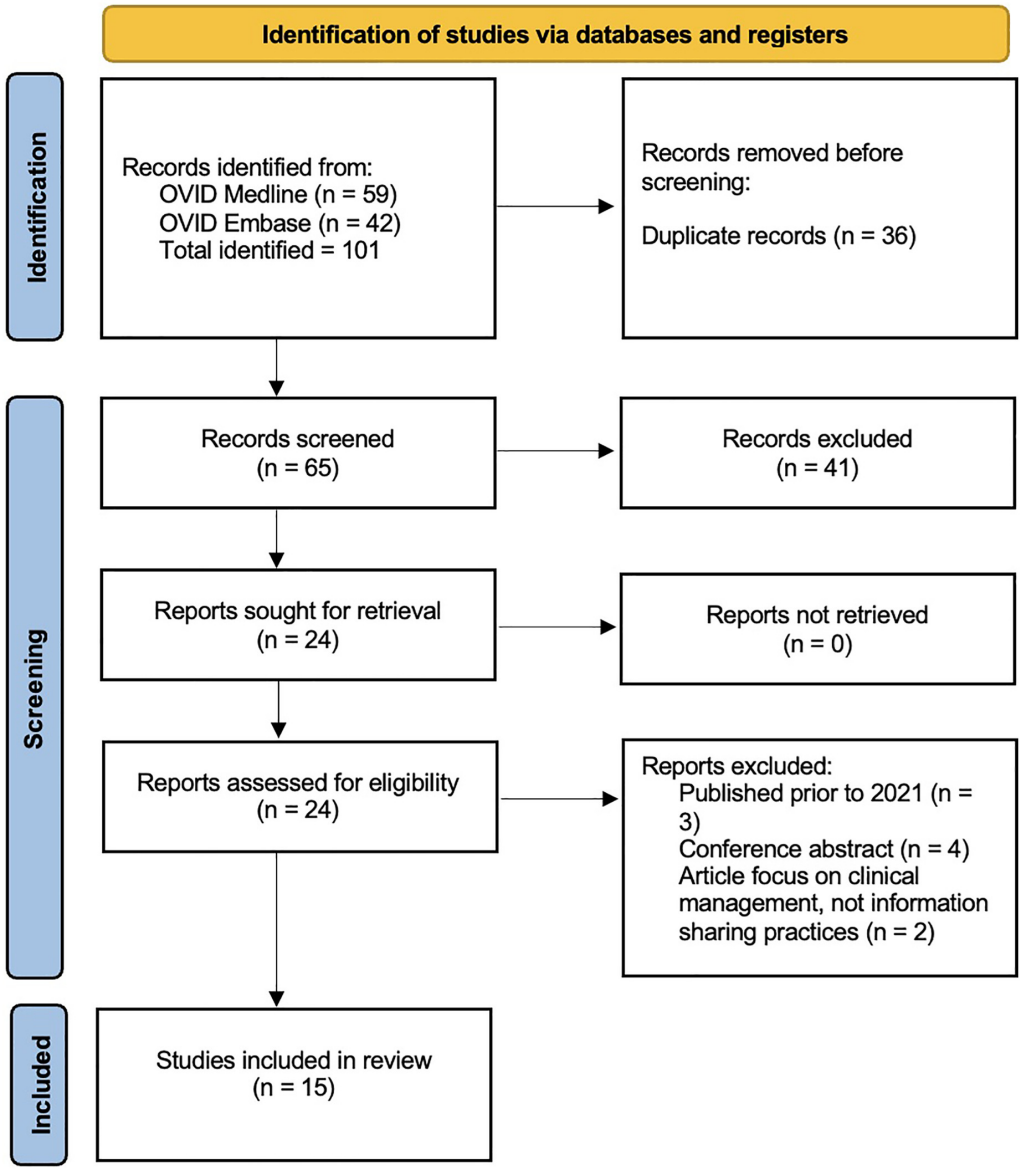
PRISMA diagram.

All articles identified by the initial search were screened for duplicates. The resultant collated list of articles had the title and abstract independently screened by J Peterson and C Graham. Those articles not relevant to the research question were excluded. This process was cross-checked using an inter-reliability assessment tool (Kappa) to assess the inclusion/exclusion decision-making for all studies identified in the initial search. This resulted in a list of articles that fulfilled the inclusion criteria from the title and abstract. These articles were then retrieved and reviewed in full, with another Kappa assessment being conducted on all full articles reviewed. Reasons for exclusion of studies following full text review are provided in Supplementary File S2. The study selection process is summarised in the PRISMA flow diagram (Figure 3). A third reviewer (DM Smith) was available in the event of any disagreements.

Once the included articles were identified, data were extracted by J Peterson and C Graham. Data on study setting, sample size, country study was conducted, study design, outcome measures, findings, conclusions were collected using an electronic form. Analysis of the extracted data was performed using a thematic synthesis approach.

### 2.6 Quality assessment

All studies included received a quality score as determined by two study team members (JP and CG) using the Quality assessment with diverse studies (QuADS) tool. The QuADS tool can be used to determine the methodological and reporting quality across a range of mixed- and multi-methods studies in health services research (17). A QuADS score was independently assessed by two reviewers (JP and CG). A Kappa score was calculated for the QuADS scores to ensure inter-rater reliability with regard to how quality is being assessed by the reviewers.

### 2.7 The analysis process

The included studies were read in full by JP and CG. Included studies underwent line by line analysis to identify key concepts for coding. Once all the included studies were coded, the data was reviewed to inductively identify themes and sub-themes around what information and how information is being shared between perinatal professionals and parents. The final written report provides a narrative evaluation and interpretation of the identified themes, outlining current strengths and weaknesses. Identification of the weaknesses in current periviable discussions will guide development of further future research for how to improve these important pre-birth conversations between perinatal professionals and parents. The final report has been written in accordance with PRISMA guidelines.

## 3 Results

The initial search identified 101 articles (Figure 3). Following removal of duplicates and screening against the inclusion/exclusion criteria, 24 articles were identified for full-text review. Of these 24 articles, nine were excluded after full-text reading as they did not fulfill the inclusion criteria [published prior to 2021 (3 articles); conference abstract (4 articles); article focus on clinical management, not information sharing practices (2 articles)]. All excluded articles and reasons for exclusion can be viewed in Supplementary File S2. This resulted in 15 articles being included in the final review (Figure 3). Within the included articles there are three articles with the same first author (Table 1; Haward et al. (18–20). For clarity in the remainder of this review, the article code from Table 1 will be provided in brackets in cases where an article by Haward is discussed.

**Table 1:** Article summary table.

Code	Title	Year	Publication	Author	Country	Study type	Scope	Summary
A	A Qualitative Study of Parental Perspectives on Prenatal Counseling at Extreme Prematurity	2022	The Journal of Pediatrics	Sullivan, Anne. et al	Boston, USA	Semi-structured interviews	To determine parental preferred language, terminology, and approach after prenatal counselling for an anticipated extremely preterm delivery.	Parents admitted at 22 + 0 to 25 + 6 weeks gestation participated in a semi-structured interview after receiving antenatal counselling. Interviews explored preferred language and decision-making approaches. Thirty-nine interviews were conducted representing 28 total prenatal consults. Key findings: • Analysis identified two overarching themes impacting the counselling experience: the need for reassurance and compassionate communication.
B	Antenatal consultation and deliberation: adapting to parental preferences	2023	Journal of Perinatology	Haward, M. et al.	USA	Open ended interviews	To analyse and compare perspectives on antenatal consultation and decision-making from a range of participants. Participants included parents with varying degrees of prematurity experience, lay persons with personal experience of prematurity through friends/family and clinicians.	80 open ended interviews using thematic and mental models analysis. Mixture of lay and parents with lived experience (including 20 with experience of bereavement). There was marked variation in the experiences of respondents. Of note, a large proportion of respondents did have not any personal/lived experience with extreme preterm birth (little explanation for the inclusion of this subgroup). • Useful insights into the need for balance in personalising information provided and using appropriate statistics and outcomes to guide discussions. • The study also provides evidence that parents desire peer support (through the incorporation of previous parent experiences and accounts) during the decision-making process.
C	Assessing shared decision making during antenatal consultations regarding extreme prematurity	2023	Journal of Perinatology	Ding, et al	Canada	Retrospective medical notes review	To assess whether antenatal decisions about care at birth for extremely preterm infants are more likely to be made when using shared decision-making (SDM) style consultations compared to standard consultations.	Prospective cohort study within a single centre perinatal unit. Retrospective chart review for all pregnant women presenting to obstetric triage between 22 + 0 and 25 + 6 weeks gestation between Sept 2015 and June 2018. There had been implementation of a new clinical practice guideline promoting SDM use within antenatal consultations in 2015. SDM consultations were those that met all four predetermined criteria: (1) Parents informed that decision is to be made and their opinion is important; (2) Options (survival-focused care or palliative care) are explained; (3) Parental preferences discussed and/or parents supported in deliberation; and (4) Decision made or deferred, and possible follow-up discussed. 217 medical notes were reviewed; 137 received antenatal consultations with 82 (60%) receiving and SDM approach. Management at birth decisions were usually made after the consultations in 88% of cases (120/137). There was no significant difference in management option chosen between consultation style [RR 1.08, 95% CI (0.95-1.26), p = 0.28]. • The authors had a predetermined definition of what communication elements would constitute an SDM approach. This definition was pre-agreed and based on the literature around SDM models. • The study was limited as this was a retrospective review of medical notes and therefore, the authors were reliant on the quality of the documentation. • Additionally, despite using the predetermined definition of SDM, it was not possible to ascertain if the SDM approach was acceptable/beneficial for parents. Also, the provided definition of an SDM approach was diffuse, making it problematic to be clear exactly how SDM differed from the standard counselling approach.
D	Bereaved Parents: Insights for the Antenatal Consultation	2021	American Journal of Perinatology	Haward	USA	Semi-structured interviews	The study aimed to explore experiences of extremely preterm infant loss in the delivery room and perspectives about antenatal consultation.	Interviews with 13 parents, reflecting on 17 pregnancies, who had experienced death in the delivery room. The article did not stipulate when the deaths had occurred or the time interval between the death and the research interview. The gestational age of the infants of the bereaved parents varied which makes drawing conclusions about the extent and quality of the specific information shared and management options presented to the parents difficult as this will vary in part depending on the gestation age of the infant. Key Findings: • Participants highlighted the need for personalized information, individualized approaches, and affective support. • Useful identified themes from the interviews included the parental desire to be a ‘good parent’ and the role professionals could have in supporting parents to achieve this. • Recruited parents also reported that ‘good’ communication from professionals should involve “placing the information in context and humanising the experience”.
E	Decision making at extreme prematurity: Innovation in clinician education	2022	Seminars in Perinatology	Sullivan, A. et al	Boston, USA	Review	Review article providing a detailed delineation of the key issues in extreme preterm decision-making and educational initiatives and future research areas needed to address these.	Narrative review with no clear rationale for how themes and references had been determined for inclusion. Key findings: • “Physicians poor at identifying which decision-making style parents prefer”. Idea of “optimal decision-making”, rather than a right answer and ‘humility’. • “Furthermore, the art of helping families articulate and construct their personal values and preferences is rarely taught”. • “Rather than acquiring a predefined skill set, future training programs need to view the transfer of skills as continuous in both learner and the environment”.
F	Decision-Making for Extremely Preterm Infants: A Qualitative Systematic Review.	2022	The Journal of Pediatrics	Krick et al	USA	Review	To synthesize and describe important elements of decision-making during antenatal consultation for threatened preterm delivery at the margin of gestational viability.	Data sources including PubMed, EMBASE, Web of Science, and CINAHL Plus were searched. All qualitative literature published on decision-making from 1990 to July 2021 was included. Twenty-five articles incorporating the views of 504 providers and 352 parents were included for final review. Key findings: • Examples of clinicians determining infant ‘best interests’ and clinician perception that parents may not be best placed to make the decision given their emotionality. • Clinicians reporting that to minimise any bias they bring the discussion they may use statistics. However, using statistics in these circumstances can carry their own bias depending on which statistics are selected to present and how.
G	Evaluating parental perceptions of written handbooks provided during shared decision making with parents anticipating extremely preterm birth	2022	The Journal of Maternal-Fetal and Neonatal Medicine	Mardian et al	Ottawa, Canada	Semi-structured interviews	To explore parental perceptions of written handbooks provided to them during antenatal counseling for anticipated extremely preterm birth.	Single tertiary level neonatal centre in Canada. Handbook developed by a working group exclusively comprised of professionals and then feedback from parents was sought afterwards. Eleven parents were interviewed about their experience using the handbook. Ten interviewed whist awaiting the birth, one interviewed after the birth. This represented only seven infants and whilst the included gestational ages were from 22 + 0 to 25 + 6 weeks, most parents had experienced birth >24 weeks (five infants); there was one 23 week infant and zero infants at 22 weeks (unclear what GA the remaining infant was). Key Findings: • Parents reported that provision of the handbook after consultation with the neonatologist was ‘ideal timing’. • SDM approach results in high-quality decisions that are informed by medical evidence and align with family values.
H	Fifteen-minute consultation: Outcomes of the extremely preterm infant (<27 weeks): what to tell the parents.	2022	ADC E&P	Yeoh et al	Australia and UK collaboration	Review	‘Best Practice’ approach aiming to outline what information should be discussed with parents facing delivery <27 weeks gestation	Practical guide outlining an approach to pre-birth conversations between perinatal professionals and parents facing extremely preterm birth. • Useful flowchart detailing the recommended approach to this conversation. • Minimal delineation of the differences between this conversation for periviable (22 + 0 to 23 + 6 week) infants and infants delivering >24 weeks. For example, the article does states that ‘death in the delivery room may occur’. However, there is little discussion around this point and no discussion of the choice between survival-focused care and comfort care that needs to be made for the extremely high risk infants ie 22–23 weekers.
I	German obstetrician’s self-reported attitudes and handling in threatening preterm birth at the limits of viability	2023	Journal of Perinatal Medicine	Schneider et al.	Germany	Professionals Survey	To evaluate obstetricians attitudes, practices and antenatal parental counseling regarding threatened preterm birth in Germany.	Online anonymous survey of 543 obstetricians in tertiary perinatal centres in Germany. Received 310 responses (57%). Key Findings: • Joint counseling with neonatologists is widely accepted. • The size of the perinatal center influenced the practical approach to threatened preterm births. • Respect for parents’ decision-making autonomy regarding the child's treatment options was important and influenced management approach at birth. • Highlighted need for future research to include perspective of multiple perinatal professional groups.
J	Gestation-Based Viability-Difficult Decisions with Far-Reaching Consequences	2021	Children	Thomas et al	Canada	Review	Summary article of the evolving nature of periviable birth management and outcomes.	Article outlining how the definition and approach to periviable birth has progressed over time. Key quotes: • Onus is with the professional to provide patients and surrogate decision-makers information that is understandable and complete, highlighting areas of medical uncertainty. This is important in forming competent collaborative partnerships with families in care and decision-making. • Antenatal and postnatal counseling at the margins of viability should take into account current limitations with the assessment of gestational maturity based on first trimester ultrasounds. This limitation should be disclosed at counseling.
K	How do Clinicians View the Process of Shared Decision-Making with Parents Facing Extremely Early Deliveries? Results from an Online Survey	2024	American Journal of Perinatology	Kim et al	USA	Professionals Survey	To better understand how neonatology and maternal–fetal medicine physicians approach the process of shared decision-making (SDM) with parents facing extremely premature (<25 weeks estimated gestational age) delivery during antenatal counseling.	Literature informed professional survey. Likert scales were used for professionals to rate their own perceived efficacy in achieving the literature-informed SDM goals. The survey achieved responses from 74 maternal–fetal medicine clinicians and 167 neonatologists, with responses from across 94% of the 81 centres surveyed. Responses highlighted the multidisciplinary team nature of these deliveries and need for multidisciplinary involvement in the shared decision-making process. • Neonatology respondents reported repeat visits with parents less often than maternal–fetal medicine clinicians (<0.001) and agreed that parents were more likely to have made delivery room decisions before they counselled them (*p* < 0.001). • Respondents reported regularly achieving most goals of SDM, except for spiritual support. • Language barriers and parents having different view from the clinician were reported as the most difficult barriers to overcome.
L	Maternal Preferences for Approach and Language Use During Antenatal Counseling at Extreme Prematurity: A Pilot Study	2021	Journal of Neonatology	Arzuaga et al	Boston, USA	Parent Survey	To determine lay-public parental preferences for approaches to prenatal counselling and preferred descriptive terminology usage by providers when discussing an anticipated extremely preterm delivery.	Exploratory pilot online survey recruited through neonatal parent/family groups on social media. A total of 142 (72%) parents participated. 78% partially completed the survey and only 37% fully completed the survey. The authors acknowledge the potential for bias as 100% of respondents were mothers (no fathers completed the survey) and 88% of respondents were college educated and self-reported their ethnicity as Caucasian. Key Findings: • The importance of not speaking down to or patronising parents. • Perinatal professionals should not rush through difficult to hear information. • Support and guidance for prospective parents could come from NICU parents who have lived experience of extreme preterm birth (peer support). • When professionals used language and presented information which removed any hope, this instilled distrust between parents and medical staff as parents were aware that there are very few situations which are entirely hopeless.
M	Personalized communication with parents of children born at less than 25 weeks: Moving from doctor-driven to parent-personalized discussions	2022	Seminars in Perinatology	Haward et al	USA and Canada	Review	Overview article with recommendations and structures for having complex discussions with parents at various points along an extreme preterm journey.	This article used fictionalised case history to explore communication approaches and frameworks to improve communication with parents facing extremely preterm birth, including an adapted version of the our-HOPE framework for communication reflections. Key Quotes: • “Acknowledging and sharing their hope assures parents that clinicians are on their side. Multiple domains of hope can co-exist, appear contradictory, and evolve as new knowledge is learned. Hope, however, does not prevent honesty: a “reconciliation of hope and honesty requires skillful management of multiple co-existing hopes, played out over time, always guided by a therapeutic intent”. • “Distilling communications to ‘one size fits all’, transfers of information or decision aids disregards the multi-dimensional nature of decision making, emotions, values, outcomes, and hope. Hope that parents find ways to live with outcomes, knowing they are or were good parents, can help them heal and rewrite their story in ways that makes sense to them.”.
N	The use of projected autonomy in antenatal shared decision-making for periviable neonates: a qualitative study	2023	Maternal Health, Neonatology and Perinatology	Thorvilson et al	USA	Discourse analysis	To assess the communication strategies used by neonatologists in antenatal consultations which may influence decision-making when determining whether to provide resuscitation or comfort measures only in the care of periviable neonates.	Single centre in the Midwest, USA. Inductive thematic discourse analysis of ‘naturally occurring data’ in the form of 25 antenatal conversations around resuscitation decisions at the grey zone of viability. The study occurred between February 2017 and June 2018. • Discourse analysis of real-time audio conversations revealed that neonatologists used language that cre­ates projected autonomy of the fetus in 20 of 25 consults. • The study identified how various discursive patterns brought the fetus into the shared decision-making process as a key agent. Use of the words “strong” and “fighter” to describe the fetus attribute characteristics to them pre-birth and instil a fighting narrative, setting up the expectation that periviable birth is something to be overcome/fought against.
O	Uncertainty at the limits of viability: A qualitative study of antenatal consultations	2021	Pediatrics	Kaemingk	USA	Discourse analysis	To gain a deeper understanding of uncertainties present and neonatologists’ communication strategies regarding such uncertainties in this shared decision making.	Prospective qualitative study from a single centre in USA. Over an 18 month recruitment period, 25 of 28 women consented to having periviable (22 + 0 to 24 + 6 weeks) pre-birth conversations between them and neonatologist recorded and analysed. Inductive applied thematic analysis of the transcripts was performed. • The authors focused on the theme of uncertainty in this article, which is one of the four themes they identified in their overall thematic analysis. • Uncertainty was actively managed by neonatologists through a variety of strategies, including providing more information, acknowledging the limits of medicine, acknowledging and accepting uncertainty, holding hope, and relationship building. • “A common mistake physicians make is to assume that information is all that is needed to guide decision-making”. • “Uncertainty is not solely an uncomfortable problem to be fixed but a necessary part of the process to prepare for and acknowledge in the care of these most vulnerable patients”.

SDM, shared decision making; USA, United States of America; UK, United Kingdom.

For quality assurance, all articles were screened by both JP and CG at each stage of the review process. There was substantial agreement between the two screening authors (JP and CG) with a Cohen's K score of 0.91 indicating 91% agreement when screening the titles and abstracts and 100% agreement after reviewing the full text articles. Any disagreement was resolved following discussion. The third author (DMS) was not required to resolve any disagreements.

### 3.1 Quality assessment

**Figure 4:**
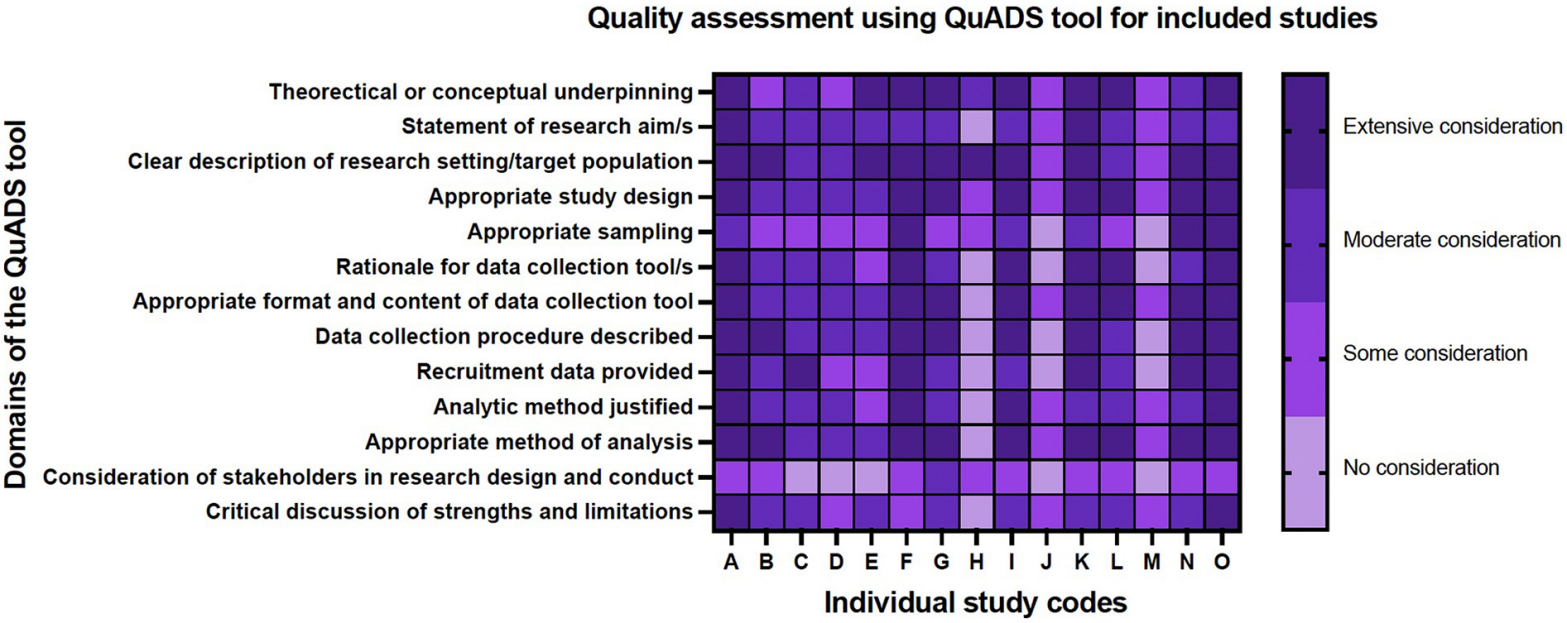
Quality assessment using QuADS tool for included studies.

Overall, the included studies were of reasonable quality (Figure 4). Most studies provided appropriate background and context for their study and had selected an appropriate methodology with which to address their research question. Several studies contained minimal information about their sampling methods, for example, the Arzuaga parent survey study (21) the authors describe using convenience sampling from social media sites with no further discussion of why convenience sampling was chosen, or in the case of some of the summary review articles, whilst the article was referenced throughout, it was unclear how the topics discussed in their reviews had been determined (22, 23). The QuADS assessment also highlights the lack of stakeholder input into the design and conduct of these studies (Figure 4).

**Figure 5:**
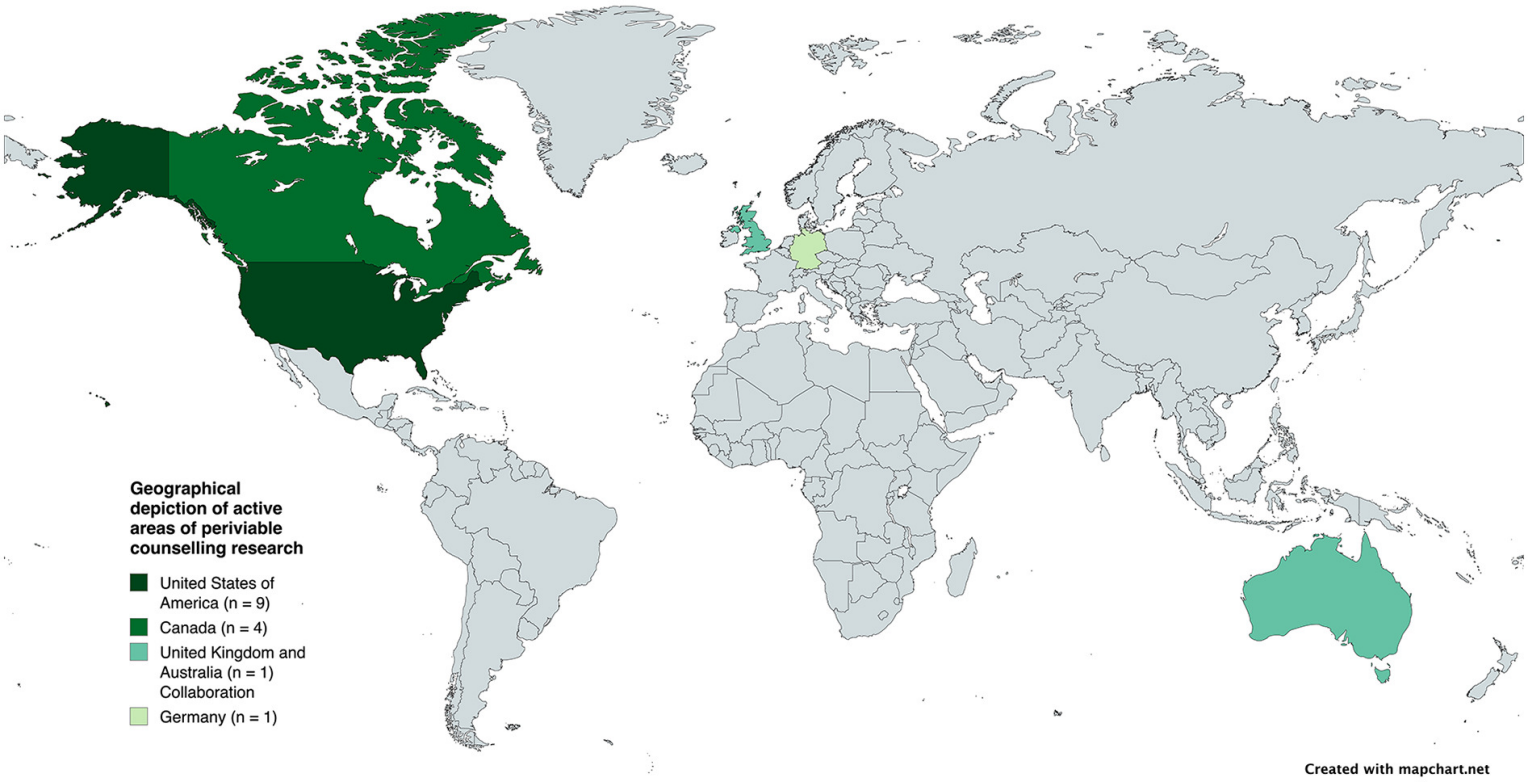
Geographical depiction of active areas of periviable counselling research.

The 15 included articles comprised a range of qualitative methods (Table 1). These included interviews with parents (four studies), review articles (four studies), online surveys (three studies), discourse analyses of conversations between perinatal professionals and parents (two studies) and one retrospective review of medical notes. The included articles were all conducted in a small number of high-income countries (Figure 5). The majority were conducted in the United States of America (nine studies) and Canada (four studies). Only two of the included studies were conducted outside North America; one from Germany (an online survey of professionals) and one publication which was a collaboration between authors in United Kingdom and Australia (‘Best Practice’ review article).

The included articles provided perspectives from a range of perinatal medical professionals and parents with varied experiences of periviable birth—including parents of surviving children and parents whose babies died. There was minimal inclusion of the perspectives of midwifery or neonatal nurses or advanced neonatal nurse practitioners. Additionally, the views of siblings, the wider family or wider clinical teams (such as general paediatricians, community paediatricians or adult physicians specialising in the long-term management of adults born extremely preterm) were not represented in the available literature.

**Figure 6:**
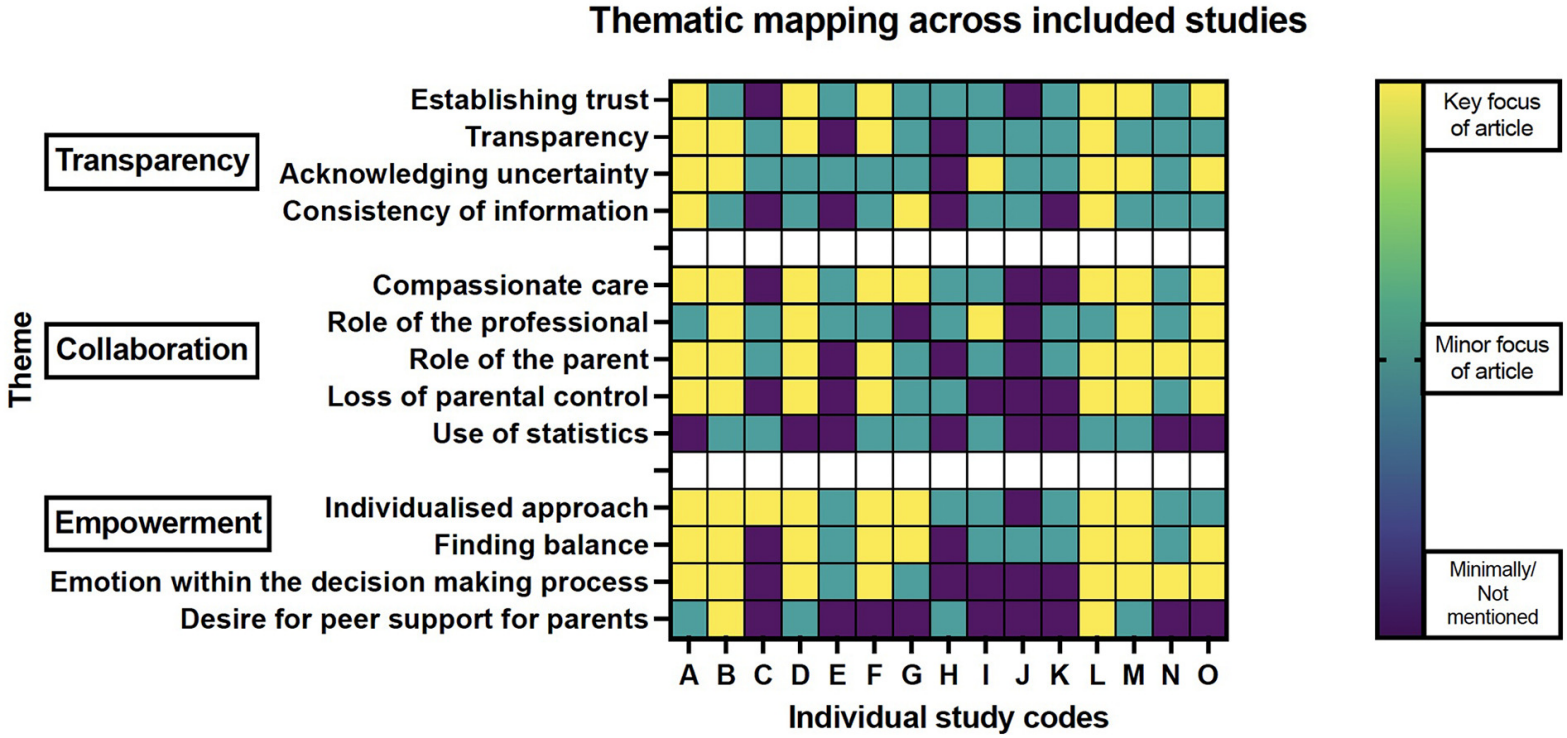
Thematic mapping across included studies.

**Figure 7:**
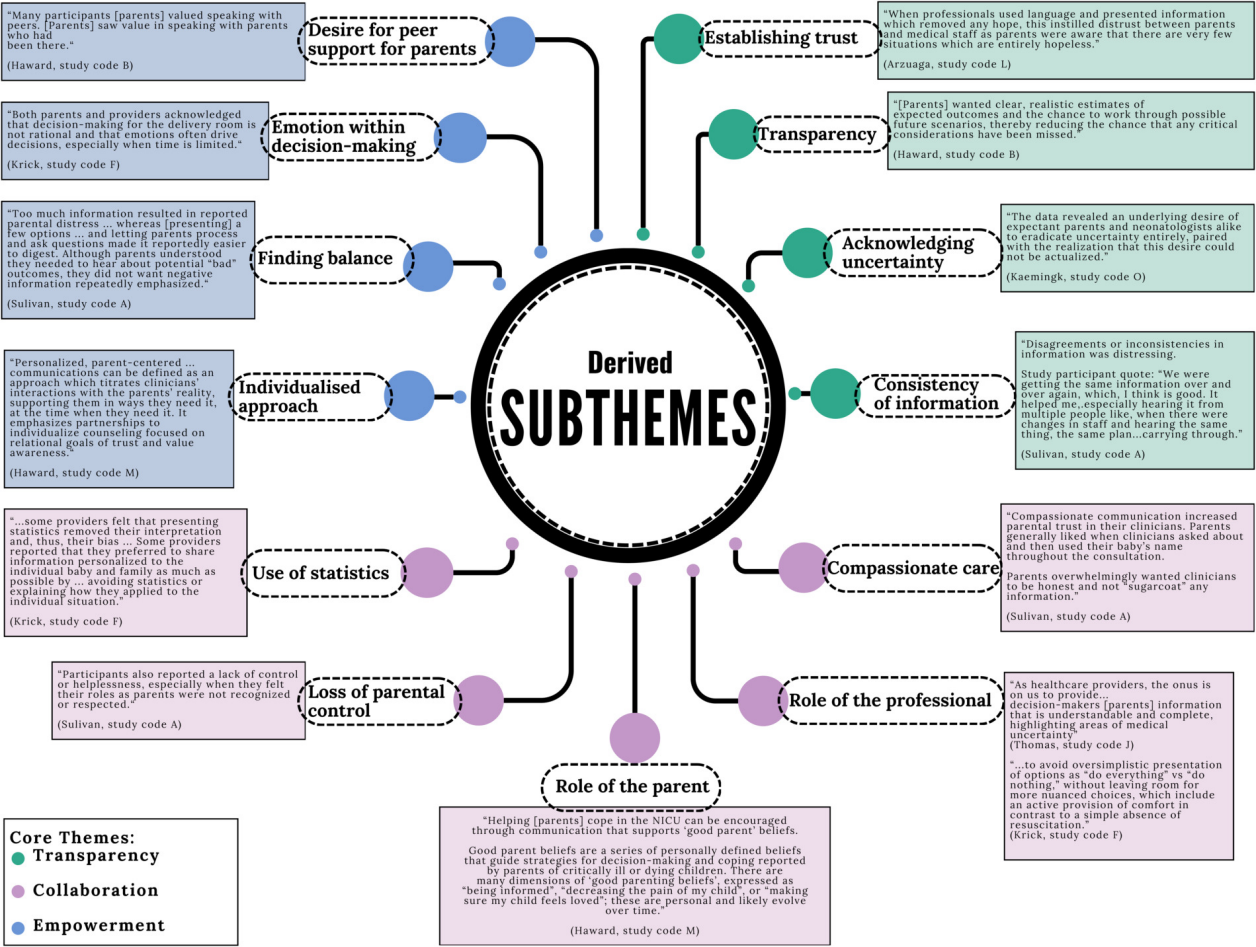
Diagram illustrating subtheme development.

The diversity of qualitative methods used by the included studies allowed exploration of information sharing approaches and practices from multiple angles and provides additional strength for the subsequent core themes identified across these studies. There were three core themes identified: Transparency, Collaboration and Empowerment. Within these themes, there were thirteen subthemes as shown in Figures 6, 7.

### 3.2 Theme one: transparency

The theme of transparency was strongly prevalent throughout all the included studies (Figure 6). This core theme was established with each study finding data illustrative of multiple facets that make up the concept of transparency; which is, to be straightforwardly perceived. In the case of transparent communication this would allude to communication which does not seek to hide, or leave out certain, relevant pieces of information. Transparent communication allows for all pertinent elements of a topic to be laid out, discussed and considered and for trust to be built between those involved in the communication. The articles in the review presented data which supported various communication tools important in creating transparency within the pre-birth periviable conversation. For example, studies reported that parents valued access to information and to feel that this information was being consistently relayed by different members of the perinatal professional team (24). This consistency increased parental confidence that the team were being open and honest with them, and this built trust (19). The data also showed that parents appreciated the need for professionals to balance the information provided and to supply this information at a pace that matched the individual parent and circumstance (24). Parents desired reassurance that when professionals were discussing different considerations of the periviable birth journey with them (such as different management options at birth), that professionals were presenting all relevant information and options to them with an open conversation about what those options could look like and what the potential implications were likely to be (23). There was an acknowledgement that this was not merely a case of presenting all information and data to the parents, as this would have the potential to result in information overload. Rather, the studies illustrate a desire from parents for information to presented in alignment with their individual circumstances and an ability to humanise the information; for example, referring to their baby's name, being aware that their baby and themselves as parents exist within a wider family and social structure, detecting and responding to the prompts and priorities of the parent in the moment, rather than a predetermined agenda set by the professional.

“Acknowledging and sharing their hope assures parents that clinicians are on their side. Multiple domains of hope can co-exist, appear contradictory, and evolve as new knowledge is learned. Hope, however, does not prevent honesty…. reconciliation of hope and honesty requires skillful management of multiple co-existing hopes”.

- **Haward, et al. (study code M)**

A crucial element within the theme of transparency was for professionals to acknowledge the inherent uncertainty that comes with periviable birth (25). It remains difficult pre-birth for professionals to predict with any accuracy which periviable infants will survive and which will die (26). The data show that professional communication which attempts to remove hope from the conversation was associated with parental distrust of the professional as parents were aware there is evidence that some periviable infants can survive and therefore, there are very few periviable birth situations which are entirely devoid of hope (21). Instead, an acknowledgement of the uncertainty by the professional was conducive to creating a balanced discussion of the clinical information and the parents’ views and values; both key elements of achieving shared decision-making (20).

Discussion of uncertainty can be difficult for perinatal professionals. The Kaemingk, et al. article (study code O) (27) conducted discourse analysis of 25 recorded pre-birth periviable conversations between neonatologists and parents. Their analysis provides evidence that uncertainty within these conversations is actively managed by professionals using a variety of strategies. Some neonatologists attempted to manage the issue of uncertainty through increased blocks of information provision within the conversation. The effect of this was for the neonatologist's voice to dominate the conversation with little to no interaction from the parents, indicating potential overwhelm or disengagement. Conversely, professionals who addressed the uncertainty through acknowledging the limits of medical knowledge, accepting the presence of uncertainty in these periviable cases and holding hope with the parents were able to demonstrate transparency and build trust, aiding the process of meaningful involvement of parents in the decision-making process.

### 3.3 Theme two: collaboration

“As healthcare providers, the onus is on us to provide.. decision-makers [parents] information that is understandable and complete, highlighting areas of medical uncertainty”

- **Thomas, study code J**

The second theme identified clearly across the included studies was that of the role that the professional and the parent occupy within the pre-birth periviable conversation and how the interaction between these roles is enacted.

For perinatal professionals their perception of their role centred on the provision of clear information to the parents about the medical components of periviable birth needed to inform the decision-making process; such as, medical information regarding mortality and the short- and long-term medical consequences of being born in the periviable period. Across the included studies perinatal professionals reported that they had the role of ensuring the rational elements of the decision-making process were being considered (19, 27, 28). Studies demonstrated that perinatal professionals report that the heightened emotional state of the parents would influence their decision-making process and prevent them from making a rational decision (29). In order to adopt this more ‘rational’ stance within the conversation, some professionals relied on a variety of techniques, such as, avoidance of the topic of uncertainty, use of statistics and avoidance of nuance within the conversation (24, 27, 29). This was demonstrated through professionals presenting management options to parents as a dichotomy, rather than a spectrum of options; for example, presenting survival focused care as ‘doing everything’ and being in opposition with comfort care which is then the necessary opposite and becomes the ‘do nothing’ option (29). This is problematic as to have an in-depth discussion of the details and relative benefits and risks of each approach requires that the clinician can give a clear explanation of the process of comfort care at delivery in order that parents are able to picture what this would involve. Full descriptions of comfort care enable parents to name and discuss what this process could look like for them and their baby. Provision of high-quality comfort care at birth is an active process that requires senior perinatal professionals with experience in bereavement and compassionate care practices: it should not be constructed as a ‘do nothing’ approach. Additionally, presenting options as a dichotomy of ‘do everything’ or ‘do nothing’ can restrict the parents access to developing an individualised care plan for delivery room management. Rather than constructing this dichotomy, it may be beneficial for some parents to discuss the options for a stepwise progression of care for their baby. This could involve relatively simple supportive interventions after birth, such as intubation and surfactant, and continuation of intensive care is assessed continuously over the following days of their neonatal journey with provision of palliative care along that process (a ‘trial of life’ approach) with the professional and parental understanding that if the infant is not responding to intensive care and the burden of care is becoming excessive that care may be reorientated; such as in the case of severe respiratory failure or, severe morbidity concerns, for example, significant bilateral intraventricular haemorrhages (29, 30). Parents need perinatal professionals to be able to move away from dichotomous presentation of options and to be able to navigate uncertainty and nuance within the pre-birth conversation, in order to develop more detailed individualised birth plans for their baby.

Professionals expressed a potential for them to experience their own biases based on their previous clinical experiences of managing other periviable infants (28). Several of the included studies showed that perinatal professionals attempted to use statistics to overcome that bias with the justification utilising statistics removed their own personal interpretation of the situation, thus providing the parents with less biased information (28, 29). The use of statistics within periviable pre-birth discussions is complex (26). Data can vary depending on the source, the denominator and the framing. For example, survival rates can vary significantly depending on if local, national or international data is selected. Therefore, the selection of which specific statistics are presented to the parents has its own potential for introducing bias and may serve to have varied impacts on the decision-making process for both professionals and parents.

Professionals acknowledged their role in supporting parents through the decision-making process. Several studies demonstrated that some professionals aim to occupy a protective role and have concerns about causing increased parental distress through the provision of ‘false hope’ (24, 29). Studies reported that professionals could find it challenging to allow room for holding hope with parents and often felt that the by emphasising the risks of being born at periviability, that this could serve to minimise any false hope (29). The issue of false hope was not one that was echoed by the parents involved in the studies in this review. Parents understood that clinicians needed to provide them with a full picture of information and that some elements of this information would be distressing to hear. However, there was a consistent finding across the studies that parents valued honest and direct information which was not “sugar coated” (24). Parents also valued practitioners who were able to discuss the more distressing pieces of information without excessive emphasis or repetition.

Parents generally expressed that their role as parent placed them in a position of decision making authority for their baby and that they viewed the clinician as a facilitator to enable the consideration of medical information alongside helping the parents work through their reflections and reactions to the medical information, allowing supported time for the parents to incorporate this new information within their personal value systems and reach the best decision for their baby and their family (18, 21, 31). Parents valued having informed and experienced professionals who were able to have an established evidence base for the information that was being shared with them but preferred that the information was personalised and specific, as much as possible, to their individual baby and family circumstances, rather than a vague or generic use of statistics (19, 27).

The experience of having a periviable birth can be an unexpected and disorientating experience for many parents. Within the included studies parents reported experiencing a sense of loss of control or a feeling of helplessness (24). Perinatal professionals can hold an important role in ensuring that their periviable counselling approach takes into consideration the strong parental desire to be a ‘good parent’ (19, 20). Research shows that for parents of critically ill or dying children and there are several dimensions which constitute the role of the ‘good parent’. These are that parents desire to be informed about their child's condition, to be able to participate in decisions around their child's care, to be able to reduce any pain their child may experience and to ensure that their child feels loved (20). The extent to which these different components are expressed at any given point in time will differ depending on the parent and the circumstances. These constituent elements of the good parent role can be actualized through the pre-birth conversation provided professionals are cognizant of the importance of these elements to parents. Clear acknowledgement of their role as parent and specific descriptions of what parents can do with their baby that address those components of the ‘good parent’ should be included in pre-birth conversations (19). These could include descriptors of how parental contact with their baby can be facilitated at delivery, importance of parental touch and voice on the neonatal unit, bonding squares, parental updates and involvement in decisions around feeding and cares. Inclusion of detailed descriptions of ensuring comfort, avoiding pain and enabling the parent to provide love to their baby can all be integrated in the pre-birth conversation and can be included whether survival-focused or comfort care approach is deemed most appropriate.

### 3.4 Theme three: empowerment

“A common mistake physicians make is to assume that information is all that is needed to guide decision-making.”

- **Kaemingk, et al. (study code O)**

The third theme identified within this review was that of empowerment. This theme brings together four subthemes centred around different aspects of the power differential between parents and professionals within these conversations. These components are: (i) the need for an individualised approach, (ii) the need for balance within these conversations, (iii) an appreciation of the role that emotion holds within the decision-making process and iv. the desire from parents for peer support during this decision-making process. Several of the studies included in the review emphasised the importance for parents that the communication they received from their professional team had been personalised to their specific circumstances (Figure 6). This made engagement with that information more accessible and established a sense of partnership between parents and professionals. This is necessary to promote trust and to facilitate discussion of the parent's values and priorities pertinent to making a decision about management at birth. Knowledge of the individuality of the parent can additionally serve to distil them in the mind of the professional as an individual autonomous person within the conversation (32).

Many of the studies commented on the need for balance within these pre-birth conversations (Figure 6). The need for balance existed across multiple levels within the conversation. This included balance within the content of the information discussed, ensuring that all relevant management options were outlined and fully discussed with parents, including options for comfort care (24, 27). As stated, the need for balancing hope alongside provision of realistic information was important to parents across the studies. Where professionals were unable to navigate this balance, there was distrust and disengagement from parents (21).

The included studies also supported the need for balance in the value placed on the respective roles of parent and professional within the conversation. Given the ethical complexity of periviable birth management decisions, it is important that parents are given facilitated time and opportunity to explore their moral values and priorities in relation to the individualised medical information provided by professionals. Within these pre-birth conversations professionals should dedicate time to facilitate exploration of the parental perspective, rather than allowing the pre-birth conversation to be dominated only with the provision of the factual medical information (20).

Six of the included studies also contained reference to a desire from parents for peer support (18–22, 24). This was generally expressed as expectant parents wishing that they had been able to access the experiences and reflections of other parents who had been through a similar periviable birth (21). It was felt that hearing from parents who have experienced periviable birth could provide expectant parents with reassurance that the overwhelm and uncertainty about the decisions that they were facing was something that had been experienced by other parents. Peer support also had the potential to provide more comprehensive information about what different periviable journeys may look like, particularly around options and elements of care that professionals can find difficult to articulate, such as the specifics of comfort care and the dying process (24).

Along these lines several of the studies commented on the role that emotion occupies within the decision-making process (Figure 6). There was an acknowledgement that professionals may feel that the heightened emotional state of the parents undermines or precludes the parent from being able to make a rational decision (28). Other studies within the review highlighted the necessary role that emotion has when making significant decisions and that emotion is a useful method through which to explore the various issues and considerations relevant to that decision (19, 24, 29). By acknowledging and discussing the emotions being expressed by the parent the concerns and hopes held by that parent and family can be better delineated and incorporation of these can result in a more individualised care plan being determined.

## 4 Discussion

This review has outlined three core themes of transparency, collaboration and empowerment that should be integrated within pre-birth periviable conversations between perinatal professionals and parents to improve the quality and impact of these discussions. This review has demonstrated that parents value clear, accurate and realistic information from perinatal professionals. There was an acceptance that parts of this information pertaining to periviable birth will be distressing and difficult to hear, but that being provided with all pertinent information was important to enabling parents to understand all management options available to them; for example, professionals providing the option for and detail about comfort care, rather than avoiding or omitting this. Parents desired accurate information tailored to their circumstances and delivered in an individualised and compassionate way. Whilst perinatal professional may not endeavour to conduct insensitive discussions with parents, some of the communication techniques described in the studies (removing hope, not acknowledging or empowering the role of the ‘good parent’, avoiding uncertainty and emotions within decision-making) which are used by professionals could prevent effective information sharing and serve to compound the trauma already being experienced by parents facing periviable birth (33).

The findings indicate that whilst these pre-birth periviable conversations are complex, there is scope to improve them through modifications to counselling approaches and professional educational strategies. These include providing professionals with techniques to be able to talk about and navigate uncertainty with parents, acknowledging the limits of medical knowledge and being able to engage with and hold space for hope within these discussions. Healthcare professional education programmes have approaches and appraisal methods which focus on the student providing the one ‘right’ answer. However, clinical medicine involves a myriad of grey-scale decisions and whilst there is professional awareness of the need for shared decision-making, this cannot be actualised without educational strategies to increase perinatal professionals’ empathy, compassion and the ability to sit with uncertainty. The evolving body of research around narrative medicine education sessions and programmes speaks to the increasing understanding and appreciation for these skills within clinical medicine (34–36). Narrative medicine is an approach which utilises art, music, poetry and writing to increase skills of listening, reflection, empathy and human connection within clinical medicine (37–39). These skills are needed to deliver individualised, compassionate care to patients and align well with the three core themes identified in this review.

**Figure 8:**
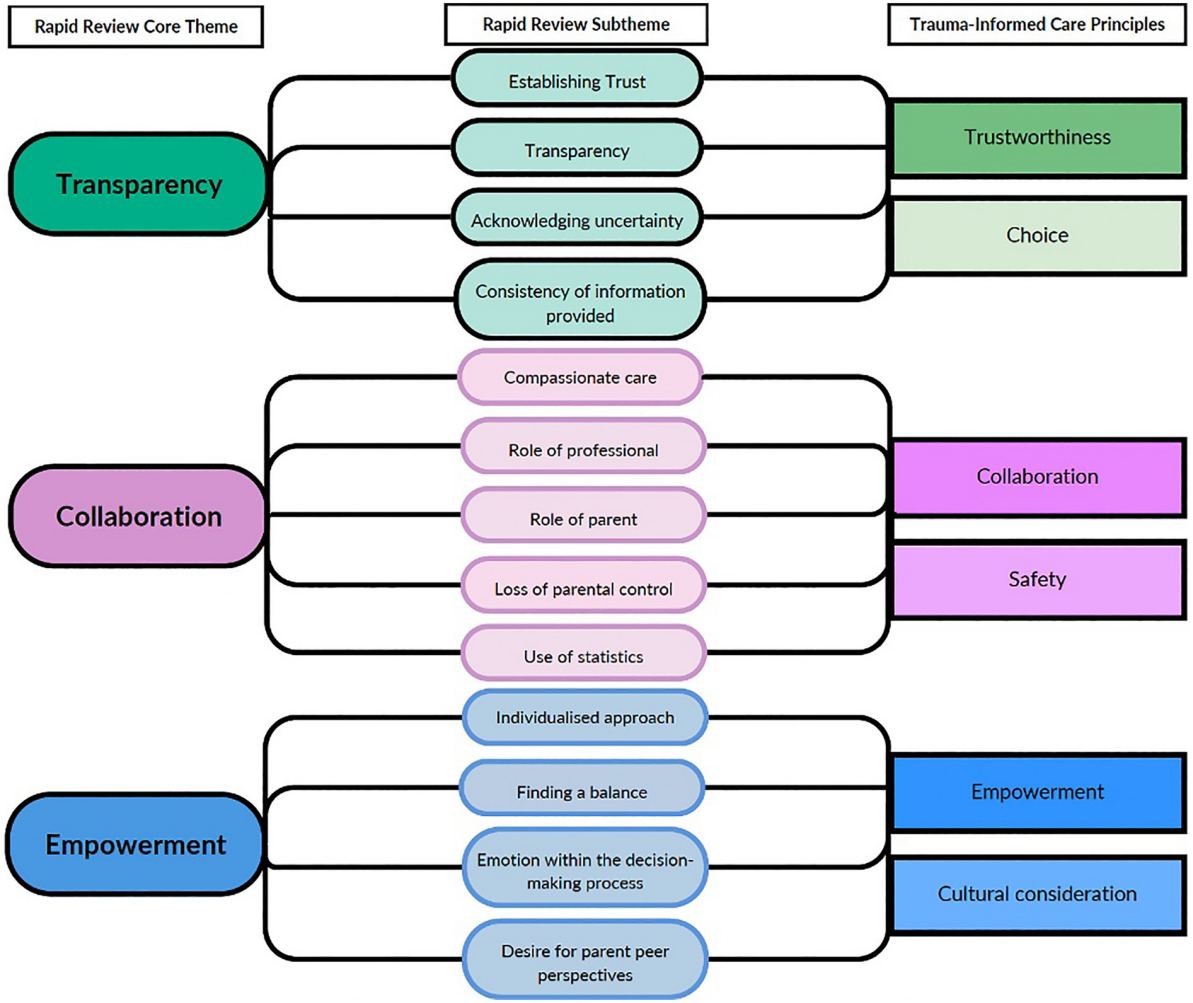
Thematic mapping to trauma-informed care principles.

Periviable birth can be a trauma-inducing experience for parents due to the significant uncertainty and loss of control that it confers over their immediate circumstances and imagined future. After presenting to hospital in threatened or confirmed periviable labour, parents encounter numerous perinatal professionals who are providing information on an evolving and uncertain labour and delivery process. Trauma can occur where there is “an event, series of events, or set of circumstances that is experienced by an individual as physically or emotionally harmful or life threatening and that has lasting adverse effects on the individuals functioning and mental, physical, social, emotional or spiritual wellbeing” (40) (pg 7). Periviable birth certainly sits within this definition. A trauma-informed approach to communication aims to recognise situations which may be traumatising, work to avoid exacerbating or retraumatising and instead to promote psychological safety, choice and control (40, 41). The findings of this review align and overlap with the six principles of trauma-informed care (Figure 8). This indicates the role that trauma-informed care approaches could have in improving the quality and experience of pre-birth periviable conversations for parents by empowering them to have access to information (control), have their perspectives and views on the information discussed and incorporated into decisions (choice) and transparency and compassion in the way the conversation is conducted (psychological safety). The need for trauma-informed care to be integrated within perinatal services is being increasingly recognised and called for (33, 42, 43). Specific guidance on trauma-informed practice from the United Kingdom's Office for Health Improvements and Disparities outlines that using this approach in clinical practice is “a means for reducing the negative impact of trauma experiences and supporting mental and physical health outcomes” (41). To successfully instil the themes identified within this review, and trauma-informed principles more generally, into perinatal clinical practice there are numerous considerations for service provision design and future research. Service provision changes may include coordination of midwifery, obstetric and neonatal team approaches to ensure clear, individualised and consistent information is conveyed to parents from each team. Services could consider co-creation of information sources for parents which include accounts from previous parents who have experienced periviable birth. This would work toward acknowledging the peer support that was desired by parents within the included studies. Services should aim to have support for perinatal professionals to prevent and address issues of burnout, compassionate stress and secondary traumatic stress that can occur through providing care to periviable infants and their families (40). Future research is needed to determine effective educational methods for ensuring perinatal professionals can navigate conceptually complex topics, such as uncertainty, within conversations with parents.

### 4.1 Strengths and limitations

The results of this review are strengthened through the systematic methods which were used to search and screen the literature. Additionally, by using a recent date range for inclusion (2021–2024) this review has focused on the latest approaches to periviable pre-birth conversations. Therefore, the findings reflect contemporary practice and increase the relevance to perinatal professionals.

This review is limited by the restricted geographical areas and cultures that the research was conducted in. All studies were conducted in Western societies, with the majority being conducted in the USA and Canada. Some of the individual studies also acknowledged that individuals who agreed to participate in the study were from a fairly homogenous ethnic and economic grouping (Caucasian, college educated, mothers) (21). The results of this review may, therefore, not represent the views and priorities of parents and practitioners from other areas of the World working within different cultures and societies and varied healthcare systems. Additionally, many of the articles included in the review included a wide gestational age range (22 + 0 to 25 + 6 weeks) in their definition of ‘extremely preterm’ (or periviable). When considering survival and morbidity risks, this creates a starkly heterogenous group of infants and outcomes. This variation in survival and morbidity outcomes would be expected to impact the pre-birth conversation. The reasons for the wide gestational age ranges are poorly described in the included studies. One explanation could be that international professional frameworks differ in their definitions and recommended approaches to periviable birth. Professional guidance from the American Association of Paediatrics recommends an individualised, holistic assessment of the infant, even at 24 + 6 weeks gestation (44). Whereas the British Association of Perinatal Medicine recommends survival-focused care from 24 + 0 weeks (3). Given that the majority of the included articles were conducted in the USA, this may account for the broad gestational age ranges within the included studies.

## 5 Conclusion

This review focused on the information sharing and communication practices for the pre-birth discussion (or ‘counselling’) between perinatal professionals and parents facing periviable birth. From the available literature, three core themes have been identified that were present across the included studies and have been demonstrated to map onto the six principles of trauma-informed care. The pre-birth periviable conversation occurs in, often unexpected, trauma-inducing circumstances for parents. Perinatal professionals involved in these conversations hold a critical role in how the narrative develops for parents around their understanding of periviable birth, what they potentially will face as a family, what their options are and what level of connection and compassion they can expect to receive from their perinatal team. Perinatal professionals need to be better equipped psychologically and educationally to understand and incorporate a trauma-informed approach to their counselling practices and the core themes identified in this review should form the basis for future research into these pivotal and nuanced conversations.

## Data Availability

The original contributions presented in the study are included in the article/Supplementary Material, further inquiries can be directed to the corresponding author.

## Footnotes

^1^This is clinically not accurate information. Intervention can be offered. The risks and benefits in each individual case should be discussed with the parents. It is not true that nothing can be offered at this gestation.
