# Correction: A rapid review of periviable (22 + 0 to 23 + 6 weeks) counselling practices and the need for a trauma-informed care approach

**DOI:** 10.3389/fped.2025.1640856

**Published:** 2025-06-23

**Authors:** Frontiers Production Office

**Affiliations:** Frontiers Media SA, Lausanne, Switzerland

**Keywords:** neonates, extreme preterm, periviable, resuscitation, counselling, trauma and maternity care

Correction on A rapid review of periviable (22 + 0 to 23 + 6 weeks) counselling practices and the need for a trauma-informed care approach By J. Peterson, C. Graham, E. D. Johnstone, A. Mahaveer, D. M. Smith. (2025). Front. Pediatr. 13. doi: 10.3389/fped.2025.1553040

Affiliation “University of Ottawa, Canada” was erroneously given as “Health Canada, Canada” for reviewer Myuri Sivanthan (Manogaran).

The original version of this article has been updated.

