# Supplementary material for: A rapid review of periviable (22 + 0 to 23 + 6 weeks) counselling practices and the need for a trauma-informed care approach
Source: Front Pediatr. 2025 May 22;13:1553040. doi: 10.3389/fped.2025.1553040 (PMC12137106; doi:10.3389/fped.2025.1553040)
Supplement: Supplementary file 1 [file Datasheet1.pdf]

PubMed results by year

Search query: ((periviable birth[Title/Abstract]) OR (22 neonate[Title/Abstract])) OR (23 neonate[Title/Abstract])	
**Year**	Count
**2024**	242
**2023**	356
**2022**	440
**2021**	408
**2020**	305
**2019**	279
**2018**	216
**2017**	244
**2016**	228
**2015**	202
**2014**	176
**2013**	192
**2012**	137
**2011**	118
**2010**	110
**2009**	101
**2008**	109
**2007**	119
**2006**	106
**2005**	84
**2004**	69
