# Supplementary material for: A rapid review of periviable (22 + 0 to 23 + 6 weeks) counselling practices and the need for a trauma-informed care approach
Source: Front Pediatr. 2025 May 22;13:1553040. doi: 10.3389/fped.2025.1553040 (PMC12137106; doi:10.3389/fped.2025.1553040)
Supplement: Supplementary file 2 [file Datasheet2.docx]

**Supplementary File S2: Excluded articles**

|  | **Title** | **Reason excluded** |
| --- | --- | --- |
| ^1^ | Antenatal counselling for parents facing an extremely preterm birth: Limitations of the medical evidence | Published prior to 2021 |
| ^2^ | Approach Speaks Louder Than Words: Parental Perspectives on Prenatal Counseling at Extreme Prematurity | Conference abstract |
| ^3^ | Comfort care plan to periviable infants: associated decision reversals and missed opportunity of antenatal steroids | Focus of article not on information sharing approaches/practices (Clinical management) |
| ^4^ | Development and Pretesting of a Decision-Aid to Use When Counseling Parents Facing Imminent Extreme Premature Delivery | Focus of article not on information sharing approaches/practices (Development of picture cards covering clinical topics such as BPD) |
| ^5^ | Evaluating a parent handbook for shared decision making for extremely preterm infants | Conference abstract |
| ^6^ | If We Treat Them, They May Live: A Single Center's Experience with Treating Neonates Born at 22 Weeks of Gestation | Focus of article not on information sharing approaches/practices (Clinical management) |
| ^7^ | Resuscitation in the "Periviable" Period-Commentary of Opposing Views | Focus of article not on information sharing approaches/practices (Clinical management) |
| ^8^ | Resuscitation selection criteria and survival at limit of viability? Score of seven | Conference abstract |
| ^9^ | Teaching Antenatal Counseling Skills via Video Conference. | Focus of article not on information sharing approaches/practices (Technical report of providing professional training via video conferencing) |

**References for excluded articles above:**

1. Janvier, A. Lorenz, J. Lantos, J. Antenatal counselling for parents facing an extremely preterm birth: Limitations of the medical evidence. Acta Paediatrica. 2012. doi:10.1111/j.1651-2227.2012.02695.x.

2. Sullivan A. et al. Approach Speaks Louder Than Words: Parental Perspectives on Prenatal Counseling at Extreme Prematurity. Pediatrics. 2022. 149.

3. Pandey, R. Comfort care plan to periviable infants: associated decision reversals and missed opportunity of antenatal steroids. J. Maternal-Fetal Neonatal Medicine. 2022. 35. 6699–6703 .

4. Guillen U. Suh, S. Munson, D. Posencheg, M. Truitt, E. Zupancic, J. Gafni, A. Kirpalani, H. Development and Pretesting of a Decision-Aid to Use When Counseling Parents Facing Imminent Extreme Premature Delivery. The Journal of Pediatrics. 2012. 160(3):382-7. doi:10.1016/j.jpeds.2011.08.070.

5. Bucking, S. Mardian, E. Morre, G. Lemyre, B. Dunn, S. Daboval, T. Evaluating a parent handbook for shared decision making for extremely preterm infants. Paediatrics and Child Health. 2019. 24, e55.

6. Akpan, U. Tumin, D. Moore, R. If We Treat Them, They May Live: A Single Center’s Experience with Treating Neonates Born at 22 Weeks of Gestation. Journal of Neonatology. 2022. 36. 311–316.

7. Bansal, S. Kaushal, M. Nimbalkar, S. Bhat, S. Resuscitation in the ‘Periviable’ Period-Commentary of Opposing Views. Journal of Neonatology. 2023. 37. 264–269.

8. Salama, H. Al Rifai, A. Mahmoud, N. Al Qubasi, M. Al Obaidly, S. Sabry, I. Ben Hadj Khalifa, O. Mousa, A. Sabouni, A. Resuscitation selection criteria and survival at limit of viability? Score of seven. BJOG International Journal of Obstetrics and Gynaecology. 2021. 128. 135.

9. Kim, A. Umoren, R. Gray, M. Teaching Antenatal Counseling Skills via Video Conference. Cureus. 2021. 13. e17030.
